# Epidemiology, treatment and outcomes of antimicrobial-resistant infections in hematopoietic stem cell transplant recipients

**DOI:** 10.1097/QCO.0000000000001206

**Published:** 2026-06-18

**Authors:** Estela Carvalho de Sousa, Monica Melchio, Malgorzata Mikulska

**Affiliations:** a Infectious Diseases Department, Hospital de São João – Unidade Local de Saúde São João; b Faculty of Medicine, University of Porto, Porto, Portugal; c Division of Infectious Diseases, Department of Health Sciences, University of Genova; d Division of Infectious Diseases, AOM IRCCS Ospedale Policlinico San Martino, Genova, Italy

**Keywords:** antimicrobial resistance, antimicrobial stewardship, Gram-negative infections, hematopoietic stem cell transplantation, novel antibiotics

## Abstract

**Purpose of review:**

To provide an overview of the current epidemiology of resistant bacterial infections in hematopoietic cell transplant (HCT) recipients, evaluate recent outcome data, and discuss available treatment strategies in this population.

**Recent findings:**

Recent studies report an increasing incidence of infections caused by multidrug-resistant bacteria among HCT recipients, with marked geographical variability and a significant impact on morbidity and mortality. Several novel agents, including new β-lactam/β-lactamase inhibitors and cefiderocol, have expanded treatment options for resistant Gram-negative infections. However, most evidence supporting their efficacy derives from studies conducted in the general population, as immunocompromised and HCT patients are frequently underrepresented in clinical trials.

**Summary:**

In recent years, growing real-world and observational data have become available specifically in immunocompromised patients, supporting treatment approaches largely consistent with those recommended for the general population, while highlighting important clinical considerations, such as empirical therapy in patients at risk for resistant infections. Ceftolozane–tazobactam is a preferred option for difficult-to-treat *Pseudomonas aeruginosa*, with combination therapy reserved for selected severe cases. Cefiderocol should be considered in specific scenarios, particularly infections caused by susceptible metallo-β-lactamase-producing organisms. For resistant Gram-positive infections, optimized daptomycin dosing is recommended. Antimicrobial stewardship strategies, including de-escalation and shorter treatment courses for Gram-negative bloodstream infections, are essential to preserve the efficacy of novel agents.

## INTRODUCTION

Patients undergoing hematopoietic cell transplantation (HCT) face high risk of infectious complications, especially bloodstream infections (BSI) during preengraftment neutropenia or graft-vs.-host disease. Infections with resistant pathogens negatively impact therapeutic choices and outcomes [1].

In recent years, several novel agents have been approved for the treatment of infections due to multidrug-resistant (MDR) bacteria, and their role has been increasingly defined in the general population [2]. However, data specifically addressing HCT recipients or patients with hematological malignancies (HM) remain limited. This review provides an overview of the current epidemiology of resistant bacterial infections and summarizes the most recent evidence on available treatment options in HCT recipients. 

**Box 1:**
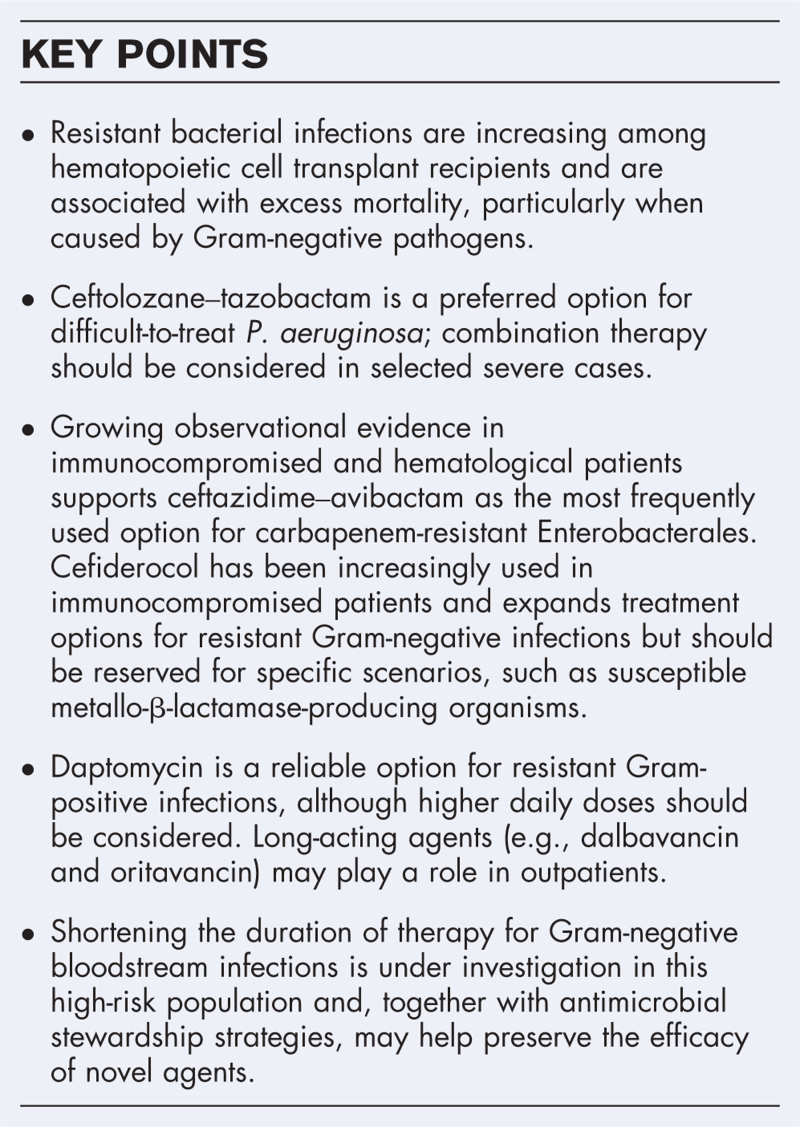
no caption available

## EPIDEMIOLOGY

A recent systematic review on European epidemiology of infections in HM/HCT patients documented BSI prevalence of 30%, with a similar rate of Gram-positive and Gram-negative bacteria (51% and 42%) [3^▪^]. Among Gram-negative isolates, resistance rates were substantial: median extended-spectrum beta-lactamase (ESBL)/third generation cephalosporin resistance (3GCR) was 30% (range 6–51%); carbapenem resistance (CR) 13% (range 1–35%; 38% among *Klebsiella pneumoniae*) and 29% (range 4–37%) of *Pseudomonas aeruginosa* (PA) BSI were due to MDR strains. Resistance rates varied markedly across centers, being higher in South-Eastern Europe, but with an overall increasing trend over time [3^▪^].

Another recent systematic review in HM patients focused on worldwide data, with approximately 20% of included studies from the United States (*n* = 53), 22% from Western Pacific WHO region, and 37% from Europe. Pooled prevalence rates of resistance were mostly higher: 21% for CR among Enterobacterales, 30% for CR among PA, and 44% for 3GCR among Enterobacterales. For Gram-positive bacterial species, methicillin-resistant *Staphylococcus aureus* (MRSA) pooled prevalence was 43% among *S. aureus* and vancomycin-resistant *Enterococci* (VRE) prevalence was 41% among enterococci [4]. The differences reflect geographical variability, possibly reflecting higher rate of VRE in the U.S. studies.

The rate of resistance among Gram-negative bacterial species varies significantly amongst European countries. In a multicenter study from Greece, 53% of BSIs in HCT recipients were caused by Gram-negative species, and half of them (38/75) were MDR, with CR pathogens being even more frequent (21%) than ESBL-producers (9%) [5].

There is limited data regarding epidemiology of infections after HCT in low-middle income countries. In a study from Argentina in HM patients, Gram-negative bacteria predominated (63%), and in HCT recipients 25.5% of BSIs were attributable to MDR Gram-negative pathogens (17.4% ESBL, 12.9% *K. pneumoniae* carbapenemase (KPC) producing Enterobacterales), while MDR Gram-positive were rare [6]. Similarly, high rate of Gram-negative BSI (55%) and high resistance rates (among *K. pneumoniae* ESBL 55% and CR 34%) were reported from HCT recipients in South Africa [7].

Data on epidemiology often included heterogeneous HM populations, therefore resistance rates among HCT recipients might have been underestimated. Indeed, in some studies, HCT was associated with a higher rate of resistant pathogens, likely due to the multiple prior courses of antibiotic therapy that these patients were exposed to [8].

Although many centers reported an increase in MDR infections overtime, reduction has been reported in some, possibly related to limiting antibiotic exposure through prophylaxis or antimicrobial stewardship [9,10]. For instance, in pre-engraftment BSI in allogeneic-HCT recipients transplanted in three periods (2004–2009, 2010–2015, and 2016–2021), there was a reduction in CR Gram-negative bacteria from 22% to 12.6%, while resistance to 3GC remained stable (40.3%), and the proportion of VRE increased from 4% to 23.5% [11^▪^].

The summary of the data in Table 1 highlights heterogeneity also in reporting resistance rates which does not allow for easy comparisons of epidemiology and potential impact of antibiotic strategies used. Therefore, we recommend reporting clearly the rate of the main resistance patterns among all Gram-negative bacteria and among the main groups such as Enterobacterales and *P. aeruginosa*.

**Table 1:** Antimicrobial resistance rates reported in major epidemiological studies performed in patients with hematological malignancies and published between June 2024 and January 2026

		Gram negatives			Gram positives	
Study, year of publication and country	Rate of Gram negative/rate of Gram positive	ESBL/3GCR	CR	*P. aeruginosa*	MRSA	VRE
Bacelli, *et al.* 2024, Europe [3^▪^]	42% (range, 13–67%)/51% (range, 26–79%)	30% (range, 6–51%) Netherlands: 7% Spain: 26% (range 6–30%)	13% (range, 1–35%) 38% (range, 18–58%) among *K. pneumoniae* Netherlands: 12% Spain 25% (range 9–32%)	CR 26% (range, 23–30%) MDR 29% (range, 4–37%)	3% (range, 1–5%) 26% (range, 21–80%), among *S. aureus*	1% (median, 1–4%) 6% (range, 2–16%), among enterococci
Sallah, *et al.* 2025, Worldwide [4]	NA	44%, among *Enterobacterales*	21% among *Enterobacterales*	CR 30%	43%, among *S. aureus*	41%, among enterococci
Palla, *et al.* 2025, Greece [5]	52.8% / 47.1%	9%	21% KPC 16% MBL 5%	NA	NA	NA
Herrera, *et al.*, 2024, Argentina [6]	60.3% / 41.9% (polymicrobial 6.6%)	17.9%	12.9% of KPC among *Enterobacterales*	MDR 7%	2%	0.8%
van Leeuwen, *et al.* 2025, South Africa [7]	55% / 38%	55% ESBL among *K. pneumoniae* 42% ESBL among *E. coli*	34.2% CR among *K. pneumoniae*	CR 3%	17%	NA
Falcó-Roget *et al.* 2025, Italy [11^▪^]	38.2% / 58.8%	40.2%	12.6%	NA	NA	23.6% among enterococci

CR, carbapenem resistance; ESBL, extended-spectrum beta-lactamase; 3GCR, third generation cephalosporin resistance; MDR, multidrug-resistant; MSRA, methicillin-resistant *Staphylococcus aureus*; NA, not available.

## TREATMENT AND OUTCOMES OF RESISTANT INFECTIONS IN HCT RECIPIENTS

Recent data confirmed increased mortality associated with MDR infection. In a review of 274 studies performed in HM population, 81 studies reported the effect of antimicrobial resistance on mortality, and 65% of them documented increased all-cause mortality in patients with resistant vs. susceptible infections; with reported hazard ratios ranging from 1.72 to 8.96 [4].

Also in a Greek case–control study performed exclusively in HCT recipients, the 28-day mortality rate was higher in patients with MDR Gram-negative BSI (28.9%), compared to patients with non-MDR Gram-negative BSI (16.2%) and Gram-positive BSI (4.4%) [5]. Negative impact of CR Gram-negative BSI associated with a twofold increase in odd ratio of mortality was also reported in a mixed cohort of HM/HCT patients from Argentina [6].

Inappropriate empirical therapy has been clearly associated with higher mortality in Gram-negative BSI [12–14].

**FIGURE 1:**
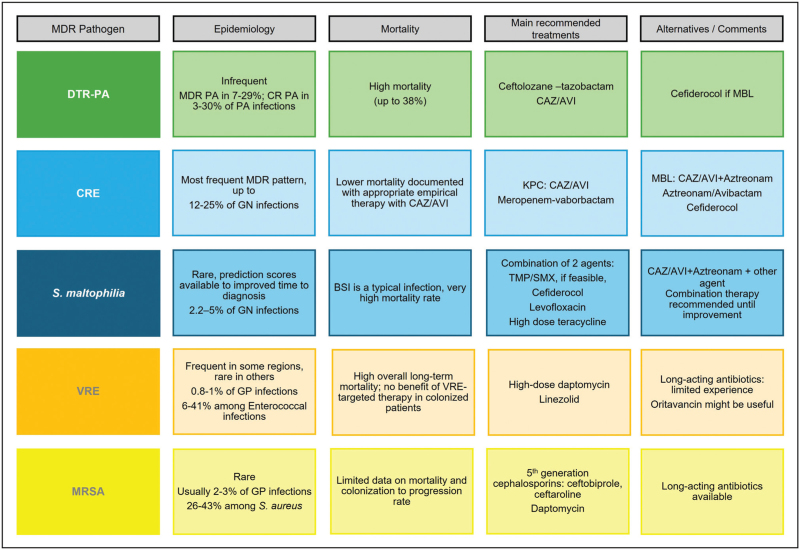
Schematic summary of incidence, mortality, and therapeutic options for the major antibiotic-resistant pathogens in hematopoietic cell transplant recipients. CAZ/AVI, ceftazidime–avibactam.

The main studies and their key findings on the treatment of the main MDR pathogens are discussed below and summarized in Table 2. Treatment of CR *Acinetobacter baumannii* infections, which are rare in HCT setting, is excluded. Recommendations provided by updated international guidelines for the general population (IDSA 2024, ESCMID 2022) [2,15] and for patients with HM by European Conference on Infections in Leukemia (ECIL 2024) [16] are provided. Figure 1 summarizes the main characteristics and treatment options for the pathogens discussed.

**Table 2:** Studies on novel agents used for treatment of MDR infection in immunocompromised patients, including HCT receipts and patients with hematological malignancies

Author, year	Type of study / objectives / timeframe	Study drug / comparators	Pathogen	Immunocompromised Population included	Population	Mortality	Other results / outcomes	Comments
Bergas A. *et al.*, 2022 (ZENITH) [17]	Multicenter, international, matched-cohort. Real-world C/T comparison	C/T vs. other treatment (most frequently: pip/tazo, meropenem or other beta lactams + AG)	*P. aeruginosa,* 91% MDR	All neutropenic HM patients	44 cases vs. 88 controls	Reduced 7-day with C/T: 6.8% vs. 34.1% (*P* = 0.001); Reduced 30-day with C/T: 22.7% vs. 48.9% (*P* = 0.005)	Lower need for mechanical ventilation on C/T arm (13.6% vs. 33.3%; *P* = 0.021)	No time to treatment provided
Shields RK *et al.*, 2025 (CACTUS) [18^▪^]	Multicenter retrospective observational; 2016–2023	C/T vs. CAZ/AVI	MDR *P. aeruginosa*	20% immunocompromised; 1.7% HCT	420 patients (210 per arm) 83% with pneumonia, 17% BSI	No significant differences	Higher clinical success with C/T (aOR 2.07); no difference in 90-day recurrence 26% vs. 35%; Emergent resistance 22% vs. 23% - no significant differences	Time to treatment approximately 72 h in both arms Differences driven by lower rate of recurrence among patients with pneumonia treated with C/T, despite suboptimal (mainly 1.5 g q8 for pneumonia) dose used in 20% of patients
Sastre-Escolà E. *et al.*, 2025 (TARZAN) [19]	Multicenter retrospective; 2017–2022	CAZ/AVI	CR Enterobacterales Resistance mechanisms: KPC 52%; OXA-48 28%; VIM 7%	All neutropenic onco-hematologic patients	54 BSI	7-day: 11%; 30-day attributed to infection: 16.5% (all cause 24%)	70% received adequate therapy in <24 h; 62% initial empirical combined therapy with AG	In 62% combined EAT; Nephrotoxicity 15% (combination with aminoglycoside/colistin); empirical monotherapy often inadequate (47%)
Tumbarello M. *et al.*, 2025 [20^▪^]	Multicenter retrospective; 2021–2023	CAZ/AVI	Enterobacterales in 74.2% of MDI (KPC in 75%) CR *P.aeruginosa* in 25% MPI	All with HM HCT (49); neutropenic (161)	198 patients (132 MDI; 66 FUO)	Enterobacterales 30-day: 17.7%; 15.2% if therapy <48 h PA: 21.4% if therapy <48 h 50% if 2nd line therapy	Mortality higher with delayed therapy; relapse in 4 MDIs (1 in vitro resistance)	AE 1.1% (renal failure); septic shock e inadequate empirical therapy independent predictors for mortality
Herrera F. *et al.*, 2025 [21]	Single-center prospective; 05/2019–11/2024	CAZ/AVI ± ATM	Enterobacterales KPC 68.2% BSIs and 81.6% non-BSI; MBL 36.4% BSI and 12.2% in non-BSI OXA-48 2.3% BSI and 5.2% non-BSI MBL+KPC 6.8% BSI	HM 35.4%; HCT 13.4%; neutropenia total 26.8%	82 CR-E infections in IC patients (BSI 44; non-BSI 38)	7-day: 6.1%; 30-day overall: 19.5%; infection-related mortality: 8.5%	7-day response 90.2%; ICU 23.1%; sepsis/shock 19.5%; multiorgan failure 13.4%	Mortality risk: refractory malignancy and septic shock
Daikos GL *et al.*, 2025 (ASSEMBLE) [22]	Prospective multicenter RCT; 2020–2023	Aztreonam/AVI vs. BAT	MBL-producing pathogen Enterobacterales (10 vs. 3); *P. aeruginosa* (2 vs. 0); *S. maltophilia* (3 vs. 0)	Not reported	15 patients with MBL-producing infections (12 vs. 3 BAT)	28-day mortality: 8% (1/12) vs. 33% (1/3)	No treatment-related serious AEs	Low mortality, although limited by small sample size and a low number of BSI (5/15)
Soueges S. *et al.*, 2025 (CEFI-ID) [23^▪^]	Multicenter retrospective; 2020–2023	Cefiderocol Monotherapy in 49.1%	*P. aeruginosa* in 56% (11.7% VIM); other NFGN in 31.8%; Enterobacterales in 12%	114 IC patients: SOT or HCT (16.6%); HM (38.5%)	114 IC patients	Overall 28-day: 37.7%; PA 30.9% Other NFGN 39% Enterobacterlaes 37.5% 90-day: 52.2%	28-day success 53.3%; relapse 17.5%; resistance acquisition on day 28: 2 cases; day 90: +3	Polymicrobial infections in 20.2%
Lombardi A. *et al.*, 2025 (CEFI-SITA) [24]	Posthoc prospective analysis; 2022–2023	Cefiderocol Monotherapy in half of patients%	Overall (multiple polymicrobial infections): *A. baumannii*, 63; *P. aeruginosa*, 32; *Enterobacterales*, 19; *S. maltophilia*, 8 In IC targeted therapy for *A. baumannii*, 24 *Enterobacterales*, 12 *P. aeruginosa*, 10 *S. maltophilia*, 2; MBL, 12	84 IC (45.4%); 15.7% HM	185 total	30-day: 40.8% IC vs. 33.3% non-IC	28-day cure: PA 81%; Enterobacterales 77.3%; MBL-GN 76.2%; *A. baumannii* 42%	Half of patients treated with combination therapy of CFDC and aminoglycosides; fosfomycin; tigecycline (exception of targeted therapy of P. aeruginosa infections); polymyxins; sulbactam or ampicillin/sulbactam (A. baumannii infections)
Torre-Cisneros J. *et al.*, 2025 (PERSEUS) [25]	Multicenter, retrospective, observational, subgroup analysis; 2018–2022	Cefiderocol	*S. maltophilia*, *B. cepacia complex*, *Achromobacter spp.*	11 IC; HCT in 6 patients	34 in this subgroup analysis (20 patients with *S. maltophilia*)	28-day all-cause-mortality 30%	Clinical cure rate 70% (*S. maltophilia*)	-
Al Musawa M. *et al.*, 2025 [26]	Multicenter retrospective; 2018–2022	Eravacycline	*S. maltophilia*	24.4% IC, 7.3% neutropenic BSI 14.6%	41 patients	30-day: 31.7%	Clinical cure rate 73.2%; 4 patients (9.8%) possible AEs	65.9% received eravacyclin for regimen consolidation
Chaftari AM *et al.*, 2024 [27]	Noninferiority RCT; 10/2021–08/2023	IPM/REL (+ GP coverage) vs. SOC (+ GP coverage)	Monomicrobial: Enterobacterales in 11 patients*; P. aeruginosa* in 3 patients (low number of microbiological documented infection).	All oncologic febrile neutropenia; hematologic malignancy (64); HCT (9)	49 IPM/REL; 50 SOC	No significant differences at TOC or late follow-up	EOT response 89.8% vs. 74% (*P* = 0.042); noninferior and superior	Small number with microbiological infection (without carbapenem resistance documented); early de-escalation (median 74 h); similar AEs
Serapide F. *et al.*, 2025 [28^▪^]	Multicenter retrospective (VRE BSI); 2016–2022	Daptomycin vs. linezolid vs. tigecycline-based regimens	VRE	13.2% hematologic patients	517 VRE BSI	30-day: 32.1%	Daptomycin-based regimens associated with increased survival	Tigecycline-based regimen associated with mortality; survival factors: Pitt score <4, surgery, early targeted therapy
De Gregori S. *et al.*, 2025 [29]	Prospective PK/PD cohort; 2020–2023	Daptomycin 8–10 mg/kg vs. 11–12 mg/kg	MSSA 7; MRSA 6; *S. epidermidis* 7 (MRSE 5); *S. haemolyticus* 1; *S. capitis* MR 1; *S. lugdunensis* MS 1	Not reported	22 patients (23 IE episodes)	Not reported	Higher PK/PD target attainment with 11–12 mg/kg; no AEs	Age independently associated with AUC; limited sample size

AE, adverse events; AG, amynoglicoside; ATM, aztreonam; AUC, area under the cure; BAT, best available therapy; C/T, ceftolozane–tazobactam; CAZ/AVI, ceftazidime–avibactam; EAT, empirical antibiotic therapy; EOT, end-of-treatment; FUO, fever of unknown origin; IC, immunocompromised; ICU, intensive care unit; IPM/REL, imipenem–relebactam; MDI, microbiologically documented infection; MDR, multidrug-resistant; MSSA, methicillin-susceptible *Staphylococcus aureus*; MSRA, methicillin-resistant *Staphylococcus aureus*; NFGN, nonfermenting Gram-negative; RCT, randomized clinical trials; SOC, standard-of-care; TOC, test-of-cure; VIM, Verona integron-encoded metallo-beta-lactamases.

## DIFFICULT-TO-TREAT *PSEUDOMONAS AERUGINOSA*

PA has always represented a major concern in HCT recipients, as high mortality rates continue to be reported (up to 38%) [30]. A recent German study in HM patients with PA-BSI reported a 30-day mortality of 22%, with poorer outcomes in patients with resistant infections due to inadequate initial therapy [31]. In a multicenter cohort of neutropenic patients with PA-BSI, 33.8% presented with septic shock, and both difficult-to-treat *Pseudomonas aeruginosa* (DTR-PA) and inadequate empirical therapy were independently associated with increased mortality [32].

Current treatment strategies include new beta-lactam agents with increased activity against DTR-PA, such as ceftolozane-tazobactam, those with activity against various MDR bacteria such as ceftazidime-avibactam, imipenem–relebactam, cefiderocol, and combination treatment, mainly with aminoglycosides.

Ceftolozane-tazobactam is recommended as preferred treatment for DTR-PA, as it overcomes most nonenzymatic resistance mechanisms and has low toxicity profile [2,33]. In a real-world study of 200 patients (28% immunocompromised), ceftolozane–tazobactam was associated with higher clinical cure rates and lower nephrotoxicity than older therapies [34], while improved survival was observed in a case-control cohort of 132 neutropenic HM patients [17].

Several retrospective studies compared ceftolozane–tazobactam to ceftazidime–avibactam with conflicting results [33]. In the CACTUS study (including 420 patients, 20% immunocompromised), clinical success was higher with ceftolozane–tazobactam in the treatment of DTR-PA. Ninety-day recurrence was frequent and similar for two treatments (26% and 35%). Of concern, treatment-emergent resistance, considering colonization or recurrent infection, occurred in 22–23% of patients with initially susceptible isolates [18^▪^].

Imipenem–relebactam has shown reliable activity against DTR-PA, which is maintained even in isolates resistant to previously described agents [35]. In a small randomized trial, it was successfully used as empirical therapy in febrile neutropenic patients, whereas data on targeted therapy in HM/HCT setting remain limited [27,36].

The use of cefiderocol in immunocompromised patients was evaluated in two multicenter retrospective studies. In the French study, with MDR-PA as the main isolated pathogen (56%, including 11.7% VIM producers), outcomes were comparable to that observed in randomized trials [37,38], and early observational study in the general population [39,40], with a 28-day overall mortality of 37.7% (PA 30.9%, Enterobacterales 37.5%, other nonfermentative Gram-negative 39.1%) [23^▪^]. Similar 28-day mortality (40.8%) was reported in a posthoc analysis of the Italian CEFI-SITA study focusing on 84 immunocompromised patients who received cefiderocol, in which immunocompromised status was not associated with 30-day mortality [24]. In both studies cefiderocol was used as combination therapy in approximately half of the patients [23^▪^,^24^].

In the absence of clear evidence favoring one agent over another, ceftolozane–tazobactam should be considered a preferred option in order to preserve the broader spectrum of ceftazidime-avibactam. Cefiderocol should instead be reserved for infections caused by metallo-beta-lactamase (MBL)-producing strains [36].

Results from studies on combination therapy for DTR-PA are conflicting [41–43]. In contrast to general population guidelines, the ECIL-10 considered the poor prognosis of DTR-PA infections in neutropenic patients and supported combination therapy with a novel beta-lactam plus other agent (aminoglycoside, fluoroquinolone, or fosfomycin) in selected cases: critically ill patients, isolates with MICs near the resistance breakpoint, or uncontrolled infection source [16].

## CARBAPENEM-RESISTANT ENTEROBACTERALES

Despite carbapenem-resistant Enterobacterales (CRE) being the group of pathogens of rapidly increasing prevalence in HCT setting, data on new antibiotics in this population remains limited.

Ceftazidime-avibactam is the most frequently used option in infections by carbapenemase-producing infections and recent data are available, mainly for KPC. In an Italian multicenter study, 132 patients had a microbiologically documented infection (MDI), mostly BSI (90.1%), with isolation of Enterobacterales in 74% of the cases (75.5% of them were KPC producers). Ceftazidime-avibactam was initiated within 48 h in 54.5% of patients with MDI, and the 30-day overall mortality in patients with Enterobacterales infections was 16.7% if ceftazidime-avibactam was started within 48 h, and 50% if it was used as second-line therapy, highlighting the benefit of its upfront use in patients at risk for CRE BSI. In four patients microbiological relapse occurred (3%, all with BSI), but only in one case the strain became resistant to ceftazidime-avibactam, while the remaining three cases were susceptible and successfully treated with combination of ceftazidime-avibactam plus amikacin [20^▪^].

TARZAN, a multicenter international study in neutropenic onco-hematological patients, evaluating ceftazidime-avibactam for treatment of CRE-BSI, reported similar outcomes: 30-day overall mortality of 24%, and infection related mortality of 16.5%. In that study, appropriate empirical or targeted therapy was initiated within the first 24 h of BSI onset in 70% of cases [19]. Similar results were reported from Argentina with ceftazidime-avibactam used alone or with aztreonam for MBL- producing bacteria in CRE BSI: 30-day overall mortality 27.3% [21]. No separate outcome results were provided for KPC and MBL producing bacteria.

While meropenem–vaborbactam and imipenem–relebactam are other treatment options for KPC producing Enterobacterales [2], limited data in the HCT setting are available. The largest experience in febrile neutropenia setting comes from the single randomized trial on empirical treatment with imipenem–relebactam vs. standard-of-care, but no CR bacteria were isolated in the imipenem–relebactam arm [27].

Data on cefiderocol against CRE are available from two aforementioned studies in immunocompromised patients: 28-day clinical cure rate of 77.3% in one, and 28-days mortality of 37.5% in another [23^▪^,^24^]. Overall, very limited data are available on cefiderocol in MBL-producing Enterobacterales in HCT setting, but in the general population concerns about resistance to cefiderocol have been reported, particularly for NDM producing Enterobacterales [44,45].

For MBL producing Enterobacterales, 2022 ESCMID and 2024 IDSA guidelines recommend the use of combination therapy with aztreonam and ceftazidime–avibactam [2,15]. Although not yet formally recommended, aztreonam-avibactam is another increasingly available option, with still limited data on clinical efficacy against MBL [22], but favorable in-vitro and PK/PD results [46^▪▪^].

Overall, in the HCT setting, ceftazidime-avibactam has the most data available, followed by cefiderocol and ceftazidime-avibactam plus aztreonam for MBL-producing Enterobacterales.

Therefore, ECIL-10 guidelines provided the highest recommendation strength for KPC-Enterobacterales for ceftazidime-avibactam, followed by meropenem–vaborbactam and imipenem–relebactam, and cefiderocol. For geographical regions where OXA-48 are frequent, ceftazidime-avibactam remains the preferred option, with cefiderocol as an alternative, while for MBLs ceftazidime-avibactam plus aztreonam is the preferred treatment, followed by cefiderocol [16].

## STENOTROPHOMONAS MALTOPHILIA

*S. maltophilia* is less common in HCT recipients than CRE or PA, with incidence of approximately 2.2–5%, but is associated with very poor outcomes (mortality up 60% in preengraftment infections) and poses specific therapeutic challenges [7,47]. Its intrinsic resistance to most beta-lactams renders standard empirical regimens for febrile neutropenia ineffective, often delaying the initiation of active therapy. In a recent study including 217 patients with *S. maltophilia* infection (8.8% of HCT recipients), the median time to active treatment was three days [48].

To minimize treatment delay, the StenoScore and StenoScore2 were developed to predict *S. maltophilia* BSI in HM/HCT patients with Gram-negative BSI, based on selected clinical features and prior antibiotic exposure [49]. A recent study reported better performance for StenoScore2 (sensitivity 86% and specificity 76%), but highlighted delays time to appropriate therapy (45 h vs. 1 h for other Gram-negative bacteria) [49].

Cefiderocol has been evaluated in observational studies, some including HCT patients, showing clinical cure rates of 62–70%, with 30-day mortality of 30–37%, supporting its use in *S. maltophilia* infections [23^▪^,^25^,^50^,^51^]. However, these relatively favorable outcomes may be influenced by study-related factors (inclusion of mainly respiratory infections and potential immortal time bias) and may not be generalizable to HCT setting, since HM was associated with an increased mortality in *S. maltophilia* BSI [52]. Although rare, intrinsic nonsusceptibility to cefiderocol has been recently reported in genogroup 4. [53], and in 4% of isolates in a recent meta-analysis [54].

The clinical use of ceftazidime-avibactam plus aztreonam remains largely anecdotal [55], although in vitro analyses have identified aztreonam-avibactam as one of the most active combinations [56]. The recent randomized ASSEMBLE trial evaluated aztreonam–avibactam in patients with MBL-producing organism infections. *S. maltophilia* was isolated in three cases, and only one achieved clinical cure at day 28 [22].

Eravacycline may represent another option. In a retrospective study including 41 patients (with approximately 25% immunocompromised) eravacycline achieved a clinical cure rate of 73.2%, comparable to that reported with other agents [26]. Of note, only 14% of patients had a BSI and only 7.3% were neutropenic, limiting the generalizability of these results to HCT population.

In summary, the current management of *S. maltophilia* infections in HCT relies on combination therapy, integrating standard agents with novel beta-lactams. Given the emergence of resistance to first-line agents (15.1% to levofloxacin, 14.6% to trimethoprim/sulfamethoxazole) [54], 2024 IDSA guidelines recommend either a combination of 2 of the following: cefiderocol, minocycline, trimethoprim/sulfamethoxazole or levofloxacin, or ceftazidime-avibactam plus aztreonam. Considering also poor outcomes and limited clinical data, ECIL-10 recommended combination therapy of two agents, with trimethoprim/sulfamethoxazole being one of them, if feasible. De-escalation to monotherapy can be considered after susceptibility has been confirmed and clinical improvement is observed [16].

## VANCOMYCIN-RESISTANT ENTEROCOCCI

Reported data on epidemiology shows an overall low prevalence of VRE infections in HCT setting in most studies. A high rate of VRE infections has been reported in some U.S. centers [57], and an increasing trend in the proportion of VRE among enterococci has been described in a European center, although the overall prevalence among HCT recipients with BSI remained low [11^▪^]. Importantly, the high rate of mortality documented in HCT patients with VRE might stem from VRE being a marker of advanced disease and poor clinical conditions, and not from VRE being the attributable cause of death as in the case of MDR Gram-negative BSI (100-day mortality reported as high as 83–100% and attributable mortality 8–14%) [57–59].

Linezolid and daptomycin are the current recommended options for the treatment of VRE infection. Regarding daptomycin, increased dosage of 8–12 mg/kg/day is recommended consistent with the higher breakpoint proposed by CLSI [60], while EUCAST did not provide breakpoints as even high doses failed to achieve adequate exposure against some isolates [61].

However, recent data describing treatment outcomes of VRE-BSI in a heterogenous cohort of 517 patients (13% with HM) demonstrated that daptomycin-based regimens (8–10 mg/kg, administered as monotherapy in 72.6% of cases), were associated with improved survival compared with regimens without daptomycin (including linezolid- and tigecycline-based therapies). Tigecycline-based alternative therapy, used in 8.2% of patients, was associated with an increased 30-day mortality [28^▪^].

Acquired daptomycin-resistance has been documented, with higher rates in young, frequently hospitalized HM/HCT patients, and especially in subsequent *Enterococcus faecium* BSI episodes after previous exposure to daptomycin [62].

Finally, oritavancin, a long acting lipoglycopeptide, demonstrated potent in vitro activity against VRE, including both VanA and VanB phenotypes [63]. However, experience in treatment of VRE infections with this agent remains limited to case reports, both in the general population and in HCT setting.

## METHICILLIN-RESISTANT *STAPHYLOCOCCUS AUREUS*

MRSA is an infrequent pathogen in HCT patients, however new therapeutical options are available. Among fifth generation cephalosporins active against MRSA (ceftobiprole and ceftaroline), ceftobiprole has been approved in 2024, also for treatment of BSI [64].

Long-acting glycopeptides (dalbavancin and oritavancin) allow the treatment of MRSA infections in the outpatient setting. Despite their initial approval for skin infection, efficacy in wide off-label use (including BSI) has been demonstrated in the general population [65].

For daptomycin, several studies suggested that doses up to 12 mg/kg/day are safe and may improve efficacy and resistance prevention not only in VRE but also MRSA infections [29]. High dosage could thus be considered also in HCT recipients.

## DURATION OF TREATMENT

Shortening the duration of targeted therapy is a relatively recent approach in various infections, including BSI. However, the pivotal trials demonstrating the efficacy of short-course therapy (7 vs. 14 days) for Gram-negative BSI generally excluded immunocompromised patients [66,67]. Observational data in hematologic patients suggest that short therapy is both feasible and effective, as shown in a large prospective cohort of high-risk neutropenic patients with Enterobacterales BSI [68].

A multicenter study in onco-hematological patients has also adressed the optimal duration of therapy for PA, showing that short courses (7–11 days) were not inferior to prolonged treatment (12–21 days). However, antibiotic discontinuation was performed only after neutrophil recovery, or initial neutrophil rise [69].

Observational studies on treatment duration are prone to selection and immortal time bias, limiting their interpretability [70]. While immunocompromised patients should be included in randomized trials evaluating antimicrobial duration, carefully conducted retrospective comparative studies could also help guide clinical practice [71].

## CONCLUSION

Infections caused by resistant bacteria are increasing worldwide among HCT recipients and are associated with increased mortality. Effective therapeutic options are currently available for the most frequently isolated resistant pathogens, and their optimal use and appropriate clinical positioning are essential. Although not the primary focus of this review, the emergence of resistance even to novel agents is increasingly reported, also in the HM setting. In addition to judicious use of both old and new antibiotics, antimicrobial stewardship strategies and updated evaluations of local epidemiology including emerging resistance mechanisms remain crucial.

## Acknowledgements

*None.*

### Financial support and sponsorship

*None.*

### Conflicts of interest

*Outside the submitted work, MM has received lecture fees and board meeting fees from: Allovir, Astra-Zeneca, bioMerieux, Gilead, Janssen, Moderna, Mundipharma, Pfizer; Shionogi; Drug/study advisory board from Mundipharma, Pfizer, Shionogi; Grant to my institution from Gilead. The other authors have no conflicts of interests to disclose.*

## REFERENCES AND RECOMMENDED READING

Papers of particular interest, published within the annual period of review, have been highlighted as:

▪ of special interest
▪▪ of outstanding interest
